# Treatment Targets for Right Ventricular Dysfunction in Pulmonary Arterial Hypertension

**DOI:** 10.1016/j.jacbts.2020.07.011

**Published:** 2020-12-28

**Authors:** Sasha Z. Prisco, Thenappan Thenappan, Kurt W. Prins

**Affiliations:** Cardiovascular Division, Lillehei Heart Institute, Department of Medicine, University of Minnesota, Minneapolis, Minnesota, USA

**Keywords:** clinical trials, pulmonary arterial hypertension, right ventricle, FAO, fatty acid oxidation, IPAH, idiopathic pulmonary arterial hypertension, LV, left ventricle/ventricular, miRNA/miR, micro-ribonucleic acid, PAH, pulmonary arterial hypertension, PH, pulmonary hypertension, RAAS, renin-angiotensin-aldosterone system, RV, right ventricle/ventricular, RVH, right ventricular hypertrophy, SSc-PAH, systemic sclerosis-associated pulmonary arterial hypertension

## Abstract

Right ventricle (RV) dysfunction is the strongest predictor of mortality in pulmonary arterial hypertension (PAH), but, at present, there are no therapies directly targeting the failing RV. Although there are shared molecular mechanisms in both RV and left ventricle (LV) dysfunction, there are important differences between the 2 ventricles that may allow for the development of RV-enhancing or RV-directed therapies. In this review, we discuss the current understandings of the dysregulated pathways that promote RV dysfunction, highlight RV-enriched or RV-specific pathways that may be of particular therapeutic value, and summarize recent and ongoing clinical trials that are investigating RV function in PAH. It is hoped that development of RV-targeted therapies will improve quality of life and enhance survival for this deadly disease.

## Highlights

• RV dysfunction is the strongest predictor of mortality in PAH.
• Unfortunately, there are no effective therapies for RV failure.
• Differences between the left ventricle and RV may allow for RV-enhancing or RV-directed therapies.
• Here, we highlight the known molecular mechanisms that promote RV dysfunction in PAH and the ongoing clinical trials investigating RV function as a therapeutic target.

Pulmonary arterial hypertension (PAH) is caused by pathological remodeling of the pulmonary vasculature, which subsequently increases right ventricular (RV) afterload and ultimately manifests as RV dysfunction. RV dysfunction is the strongest predictor of mortality in PAH (1, 2, 3), and the response of the RV to PAH-specific therapy determines survival (4,5). Moreover, RV dysfunction is not solely caused by increased afterload, as some PAH patients exhibit progression of RV dysfunction despite treatment with pulmonary vasodilators (6,7). Furthermore, patients with systemic sclerosis-associated PAH (SSc-PAH) have worse RV function—and consequently increased mortality—compared with patients who have idiopathic PAH (IPAH), despite having similar severity of pulmonary vascular disease (6,8). Unfortunately, no currently available PAH therapy directly targets the RV. Therefore, there is an unmet need to combat the mechanisms underlying RV dysfunction directly to improve long-term outcomes in PAH.

In addition to PAH, RV dysfunction is prognostic in the 2 leading causes of pulmonary hypertension (PH): PH caused by left heart disease (World Health Organization [WHO] Group 2) and lung disease (WHO Group 3) (9, 10, 11). RV function predicts mortality (12,13) and is affected independent of afterload in Group 2 PH (14). Despite having less severe pulmonary vascular disease than patients with PAH, Group 3 PH patients have disproportionate RV dysfunction and poor survival (15). The presence of RV dysfunction in Group 3 PH also identifies patients at high risk of poor outcomes (11,16). Unfortunately, PAH-specific therapies do not significantly improve exercise capacity or reduce symptom burden in Group 2 and 3 PH (17). Thus, RV-directed therapy may be beneficial for these prevalent and currently untreatable causes of PH.

## Differences Between the Right and Left Ventricles

**Figure 1:**
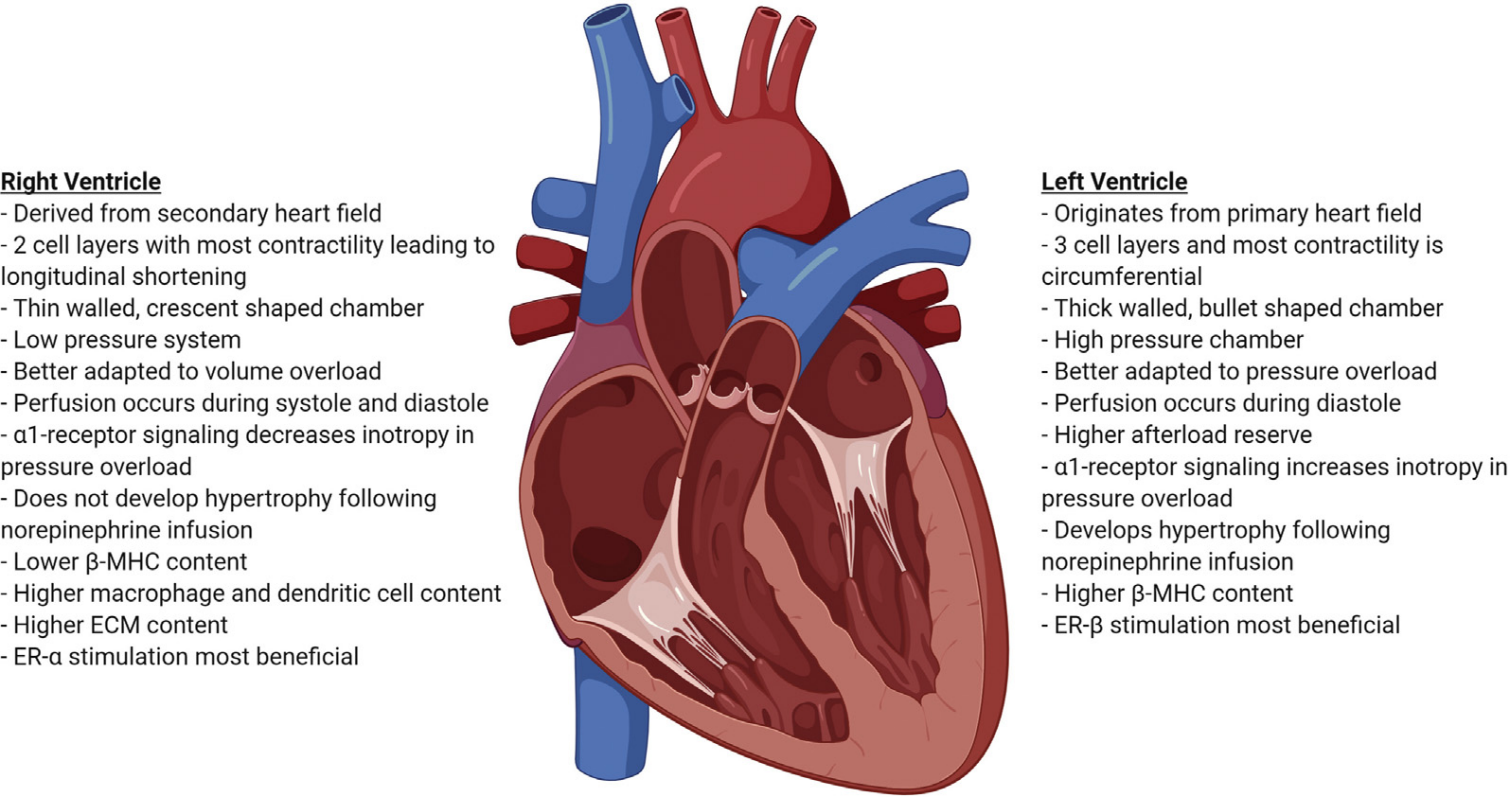
Developmental, Anatomic, and Functional Differences Between the Right and Left Ventricles ECM = extracellular matrix; ER = estrogen receptor; MHC = myosin heavy chain.

Mechanistic dissections of RV dysfunction have lagged behind our understanding of left ventricular (LV) dysfunction. This is exemplified by the fact that there are multiple therapies with proven survival benefits for LV failure (18,19) but no approved drugs for RV dysfunction. Importantly, the use of standard therapies for LV failure, such as beta blockers and targets of the renin-angiotensin-aldosterone system (RAAS), are not indicated or potentially contraindicated in patients with PAH and RV dysfunction (20,21). Thus, understanding the differences between the RV and LV may be important to define RV-directed therapies (Figure 1, Table 1).

**Table 1:** Right Ventricular-Specific or Right Ventricular–Enriched Mechanisms of Cardiac Dysfunction

RV-Specific/Enriched Targets	Description
Fibrosis	At baseline, RV has higher collagen content (75) and different expression of matrix metalloproteinases and extracellular matrix proteins (76) compared with the LV. Antifibrotic therapies that are effective in the LV (pirfenidone and eplerenone) do not reverse fibrosis in the RV (57,72).
Inflammation	RV has more macrophages and dendritic cells at baseline compared with the LV (95).
Estrogen signaling	In the dysfunctional RV, the beneficial effects of estrogen are predominately mediated by ER-α (98), but in LV pressure overload, stimulation of ER-β normalizes LV ejection fraction (106).
Ischemia	RV is perfused during both systole and diastole, whereas the LV is only perfused during diastole (31). At baseline, RV has a reduced microvascular bed with less tissue perfusion compared with the LV (135). There may be decreased angiogenesis during RV failure (110,121, 122, 123).
Epigenetics	There are baseline differences in miRNA expression between the RV and LV (140). RV-specific miRNAs that are dysregulated during RV dysfunction: miR-21, -28, -34a, -93, -126/VEGF, -127, -130a, -146b, -148a, -197, -208/Mef2c, -221, and let-7e (39,133,140, 141, 142, 143,145).

ER = estrogen receptor; LV = left ventricle; Mef2c = myocyte enhancer factor 2c; miRNA = microRNA; RV = right ventricle; VEGF = vascular endothelial growth factor.

**Figure 2:**
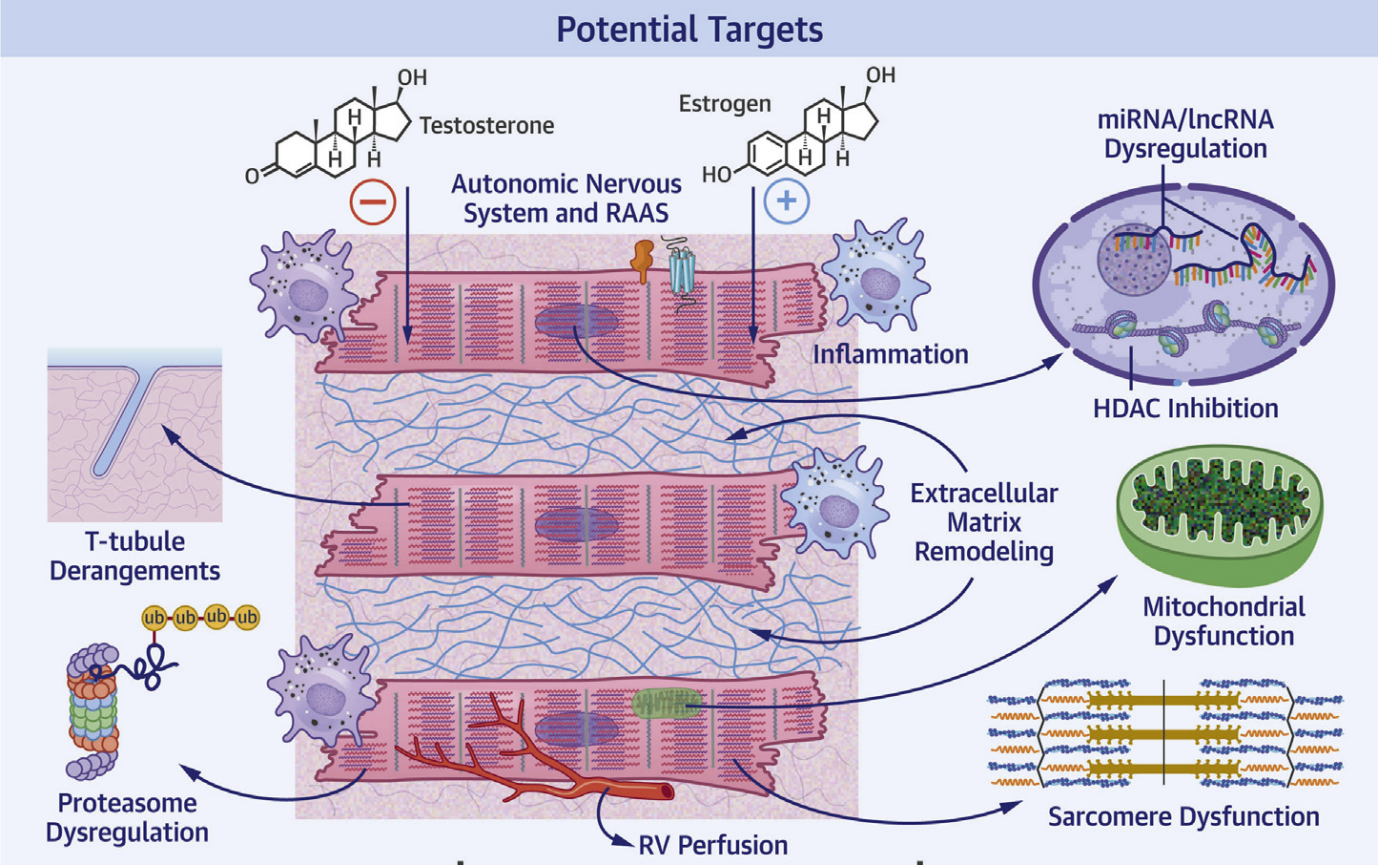
Potentially Targetable Mechanisms of Right Ventricular Dysfunction in Pulmonary Arterial Hypertension HDAC = histone deacetylase; lncRNA = long noncoding RNA; miRNA = microRNA; RAAS = renin-angiotensin-aldosterone system; RV = right ventricle; t-tubule = transverse tubule; Ub = ubiquitin.

There are developmental, anatomic, and functional differences between the RV and LV (Figure 1) that may provide insight into ways to enhance ventricle-specific function (Figure 2, Table 1). Developmentally, the LV originates first from the splanchnic mesoderm within the primary heart field (22), whereas the RV develops second from the extracardiac mesoderm within the secondary heart field (23). The prenatal RV is thick walled and generates high pressures to support fetal blood flow (24). After birth, the pulmonary circulation becomes a low pressure circuit (25), whereas the systemic circulation becomes a high pressure system, which leads to relative LV hypertrophy (26). Ultimately, the RV becomes a thin-walled, crescent-shaped chamber, whereas the LV takes a muscular bullet shape (27). Furthermore, there are dissimilarities in cardiomyocyte arrangement that lead to differences in contractility between the 2 ventricles. The adult RV has 2 layers of cardiomyocytes: a circumferential layer that brings the RV free wall toward the interventricular septum and a deeper layer of vertical fibers that result in longitudinal shortening, which accounts for 75% of the total RV contractility (28). In contrast, the LV has 3 myocardial layers. LV contraction is a function of radial fiber thickening, longitudinal fiber shortening, and oblique fiber thickening (29). Circumferential and longitudinal shortening contribute to 67% and 33% of total LV contractility, respectively (30). Moreover, there are differences in ventricular perfusion, as the RV is perfused throughout the cardiac cycle, whereas the LV is only perfused during diastole (31). Although the normal RV and LV have similar coronary flow reserves of 400% to 500% (32, 33, 34, 35), the RV has lower baseline oxygen consumption and greater oxygen extraction reserve, which lowers its risk of ischemic injury caused by decreased coronary flow (31). However, the pressure-overloaded RV is more susceptible to ischemia, with acute or chronic increases in afterload as elevated RV afterload reduces the systolic RV perfusion gradient. This curtails overall RV perfusion because the RV is normally perfused during both systole and diastole (31). Finally, there are important anatomic differences between the ventricles that alter their response to preload and afterload. The RV is thinner and has lower volume to wall surface area, which makes the RV more compliant and better adapted to initial volume increases but renders it unable to accommodate afterload increases as well as the LV (36). This is supported by a study of isolated rat hearts that shows the LV has a higher afterload reserve than the RV (37).

There are divergent molecular responses between RV and LV failure documented in animal and human studies (27,38,39). In brief, there are differences in adrenergic signaling regulation as β1- and α1-adenergic and dopamine-1 receptors are downregulated in the pressure overloaded RV, which leads to a diminished inotropic response (38,39). Although there is also downregulation of β1-adenergic receptors in LV dysfunction (40), α1-adrenergic receptors are actually upregulated and increase contractility in the pressure overloaded LV (27,38,39,41). Conversely, α1-receptor signaling decreases inotropy in the overloaded RV (27,38,39). Furthermore, there are dissimilarities in the response to catecholamines, as only the LV develops hypertrophy following norepinephrine infusion (27,38). Moreover, the shift in expression from α- to β-myosin heavy chain (MHC), which represents a fetal gene-expression pattern, occurs in both the failing RV and LV (42). However, under normal conditions, β-MHC content is lower in the RV compared with the LV (43). Finally, the response to digoxin differs in patients with RV and LV failure. Digoxin modestly increases cardiac output, decreases circulating norepinephrine, and does not change baroreceptor response in patients with PAH and RV failure (44). In contrast, digoxin attenuates baroreceptor sensitivity while also decreasing serum norepinephrine levels in patients with LV failure (45).

## Mechanisms of RV Dysfunction in PAH

### Experimental models of PAH

Several commonly used preclinical models of PAH are discussed in the following sections. Table 2 succinctly outlines the characteristics of the models, including their strengths and weaknesses. A detailed evaluation of these models was previously summarized (46).

**Table 2:** Experimental Models of Pulmonary Arterial Hypertension/Right Ventricular Dysfunction

	Monocrotaline	Hypoxia	Sugen-Hypoxia	Pulmonary Artery Banding
Method	Subcutaneous injection of 40–60 mg/kg monocrotaline	Exposure to hypoxia for 3–5 weeks	Subcutaneous injection of VEGFR2 antagonist Su5416 and hypoxia (3 weeks) and then placed in room air (≥4 weeks)	Suture or clip around main pulmonary artery
Condition Modeled	Inflammatory PAH, severe RV dysfunction	Chronic hypoxia	Pulmonary vascular endothelial injury and cellular proliferation	Increased RV afterload with proximal stenosis
RV systolic pressure (mm Hg)	40–80	30–40	60–80	60–80
Type of RV remodeling	Maladaptive	Adaptive likely due to low pulmonary artery pressures	Maladaptive	Maladaptive or adaptive depending on band tightness
Advantages	Severe PAH induced with a single subcutaneous injection, relatively inexpensive, model of severe RV dysfunction	Model of chronic hypoxia, high altitude, and chronic lung disease	Develops some pulmonary vascular changes (e.g., plexiform lesions) similar to human PAH	A model of compensated RV if band is not too tight
Limitations	Variable response, sex differences in PAH severity, can lead to myocarditis	Less severe RV dysfunction, variable response	Rat background alters PAH severity and RV phenotype	Variability in pulmonary artery banding modulates RV phenotype, highly technical, models proximal disease rather than PAH

Information obtained from references 46,121,169,170.

PAH = pulmonary arterial hypertension; RV = right ventricle; Su5416 = Sugen 5416; VEGFR2 = vascular endothelial growth factor receptor 2.

### Neurohormonal and RAAS dysregulation

A previous review highlighted the evidence of sympathetic nervous system (SNS) dysregulation in RV dysfunction in PAH, and completed clinical trials of beta blockers in PAH are largely neutral (47). Conversely, there is less known about the role of the parasympathetic nervous system in RV dysfunction. A recent study showed vagal nerve stimulation preserves RV function, although in the setting of less severe pulmonary hypertension, in the Sugen 5416-hypoxia model of PAH (48). Further studies are needed to determine if parasympathetic activation directly improves RV function.

Numerous studies show that the RAAS is involved in pulmonary vascular remodeling (49), which, in turn, affects RV function, but the direct role of the RAAS on RV function is understudied. Moreover, the role of the RAAS in PAH is complicated by the fact that angiotensin-(1-7), the product of angiotensin II metabolism by angiotensin-converting-enzyme (ACE)-2 (50), mitigates pulmonary vascular disease, leading to decreased RV fibrosis, RV hypertrophy (RVH), and RV systolic pressure in monocrotaline rats (51). Although preclinical studies show ACE inhibitors (52,53), angiotensin receptor blockers (54,55), and aldosterone antagonists (56) attenuate the development of RVH, fibrosis, and RV dysfunction, this occurs in the setting of less severe PAH, so the direct effects on the RV are not definitively understood. Finally, eplerenone has no beneficial structural or functional effects on the RV when treatment is initiated after development of pulmonary vascular disease in Sugen-hypoxia and pulmonary artery-banded mice (57).

In human studies, ACE protein concentration is increased, but there is downregulation of the angiotensin II type 1 (AT1) receptor in the failing PAH RV, likely caused by higher stimulation of AT1 receptors by angiotensin II (58). However, some ACE activity appears beneficial. For example, the ACE DD genotype, a homozygous polymorphism that increases circulating and cardiac tissue ACE activity, results in preserved RV function in patients with PAH (59). Moreover, recombinant ACE2 augments cardiac output acutely in patients with PAH (60). These findings suggest targeted activation of the RAAS may actually be beneficial for the failing RV.

Clearly, the roles of the SNS and RAAS in RV dysfunction need to be clarified. The effects of beta blockers on RV function in PAH are being investigated in ongoing clinical trials, and it is hoped that will help elucidate the role of the SNS on RV function (Table 3).

**Table 3:** Recent and Ongoing Clinical Trials Targeting Right Ventricular Dysfunction in Pulmonary Arterial Hypertension

Intervention	ClinicalTrials.gov Identifier	Pathophysiology Targeted	Study Start Date	Estimated/Actual Primary Completion Date	Estimated Enrollment	Primary Outcome Measures	Trial Status∗	Results
Carvedilol	NCT00964678	Sympathetic nervous system	06/2010	05/2014	24 (actual 10)	RVEF by CMR	Completed	Small sample size, significant improvement in RVEF from baseline (p = 0.028) (167)
Carvedilol	NCT02120339	Sympathetic nervous system	05/2014	06/2015	25 (actual 5)	RVEF by CMR	Terminated (low enrollment)	
Carvedilol	NCT01586156	Sympathetic nervous system	07/2016	06/2016	68 (actual 30)	Cardiac glucose uptake in FDG-PET	Completed	Carvedilol significantly decreased glucose uptake compared with placebo (p = 0.04) (168)
Carvedilol or empagliflozin†	NCT04345796	Sympathetic nervous system, metabolism	06/2020	05/2023	180	RV end-systolic volume index by CMR	Not yet recruiting	
CXA-10	NCT03449524	Oxidative stress, inflammation, metabolism, fibrosis	08/01/2018	12/2020	96	RVEF by CMR and PVR by RHC	Recruiting	
Anakinra	NCT03057028	Inflammation	04/2016	06/07/2018	10 (actual 7)	Peak oxygen consumption and ventilator efficiency on cardiopulmonary exercise testing	Completed	Significant improvement of heart failure symptoms (p = 0.046) (96)
Rituximab	NCT01086540	Inflammation	06/24/2011	06/05/2018	60 (actual 58)	6-min-walk distance	Completed	Not posted
Tocilizumab	NCT02676947	Inflammation	01/2016	12/18/2018	21 (actual 29)	Safety and PVR	Completed	Not posted
Anastrozole	NCT01545336	Anti-estrogen	10/2012	06/2015	18	Plasma estradiol level, TAPSE	Completed	Small sample size; anastrozole did not improve TAPSE compared with placebo (171)
Anastrozole	NCT03229499	Antiestrogen	12/07/2017	09/2021	84	6-min-walk distance	Active, not recruiting	
Fulvestrant	NCT02911844	Estrogen receptor antagonist	04/10/2017	12/05/2018	5	Plasma estradiol levels, TAPSE by echocardiogram, 6-min-walk distance, NT-proBNP	Completed	Inconclusive, small sample size (172)
Tamoxifen	NCT03528902	Estrogen receptor binder (has pro- and anti-estrogenic actions)	10/01/2018	06/30/2022	24	TAPSE by echocardiogram	Recruiting	
Dehydroepian-drosterone (DHEA)	NCT03648385	Endogenous precursor to androgens, capillary rarefaction, fibrosis, oxidative stress	01/09/2019	04/2023	24	RV longitudinal strain by CMR	Recruiting	
Ranolazine	NCT01174173	Metabolism	06/2010	01/2014	25 (actual 11)	WHO functional class, 6-min-walk test, Kansas City Cardiomyopathy Questionnaire score	Completed	Small sample size, ranolazine significantly improved WHO functional class (p = 0.0013), reduced RV size (p = 0.015), and improved RV function (p = 0.037). No significant difference in 6-min-walk distance (p = 0.09) or Kansas City Cardiomyopathy Questionnaire score (p = 0.37) (168)
Ranolazine	NCT01757808	Metabolism	08/2011	01/2015	16 (actual 12)	PVR	Completed	Not posted; safety results published in (173)
Ranolazine	NCT01839110	Metabolism	07/2013	01/2018	90 (actual 22)	RVEF by CMR	Completed	Small sample size, trend to improved RVEF with ranolazine compared with placebo (no statistics reported) (174)
Ranolazine	NCT01917136	Metabolism	08/2013	10/2018	54 (actual 21)	RV function by CMR	Completed	Inconclusive, small sample size
Ranolazine	NCT02829034	Metabolism	07/2016	12/2017	10 (actual 22)	RVEF by CMR	Completed	Small sample size with many patients lost to follow-up, trend to improved RVEF with ranolazine compared with placebo (no statistics reported) (174)
Trimetazidine	NCT02102672	Metabolism	03/2014	12/2016	25	RV function by 3D echocardio-graphy	Unknown	
Trimetazidine	NCT03273387	Metabolism	09/10/2017	11/01/2018	25 (actual 26)	RVEF by CMR	Completed	Small sample size, trimetazidine significantly improved RVEF compared with placebo (p = 0.008)
Metformin	NCT03617458	Metabolism	08/23/2018	12/2022	160 (actual 39)	6-min walk distance and WHO functional class	Active, not recruiting	
Exercise and respiratory therapy	NCT04224012	RV contractile reserve	08/2015	08/2021	96	Cardiac index by RHC	Recruiting	
Rehabilitation	NCT02579954	RV contractile reserve	08/06/2015	12/2020	60	Endurance time at 75% of maximal workout during CPET	Recruiting	
Cardiorespiratory rehabilitation	NCT03404492	RV contractile reserve	06/12/2018	04/12/2019	10	RV contractile reserve	Recruiting	

CMR = cardiovascular magnetic resonance imaging; CPET = cardiopulmonary exercise testing; DHEA = dehydroepiandrosterone; Ees = end-systolic elastance; FDG-PET = fluorodeoxyglucose-positron emission tomography; NT-proBNP = N-terminal-pro-brain natriuretic peptide; PVR = pulmonary vascular resistance; RHC = right heart catheterization; RV = right ventricle; RVEF = right ventricular ejection fraction; TAPSE = tricuspid annular plane systolic excursion; WHO = World Health Organization.

∗ Some of the trials that are active but not currently recruiting as of May 2020 may be due to the current COVID-19 pandemic.

† Trial in patients with severe functional regurgitation.

### Fibrosis

Development of RV fibrosis may initially be an adaptive process to maintain RV shape, but during prolonged stress, RV fibrosis becomes maladaptive as it increases diastolic stiffness, alters cardiomyocyte excitation-contraction coupling, and depresses myocardial contraction (61, 62, 63, 64).

It is interesting that currently available antifibrotics do not effectively mitigate RV fibrosis in preclinical studies. However, multiple preclinical drug studies show reduction in RV fibrosis is associated with improved RV function, but all occur in the setting of reduced afterload (65, 66, 67, 68, 69). Furthermore, a novel pulmonary artery debanding mouse model shows that, after pulmonary artery band resorption, RV function is restored with normalization of cardiomyocyte size and reversal of fibrosis (70).

Current antifibrotic therapies do not mitigate RV fibrosis directly. For instance, pirfenidone, which reverses fibrosis in pressure overloaded LV (71), does not reverse RV fibrosis or enhance RV function in pulmonary artery-banded rats (72). However, pirfenidone reduces RV fibrosis and remodeling in Sugen-hypoxia rats but, again, in the setting of less severe PAH (73). Second, eplerenone, which attenuates LV fibrosis (74), does not reverse RV fibrosis in Sugen-hypoxia and pulmonary artery-banded mice after PAH is established (57).

Although there are similar molecular mechanisms that trigger RV and LV fibrosis, fibrosis appears to have different roles in each ventricle. For instance, the RV can be subjected up to a 5-fold increase in afterload in PAH, whereas the LV afterload increase under pathological conditions is usually <1.5-fold (61). The heightened afterload increase in PAH may demand that RV adaptation relies more on extracellular matrix reinforcement than the overloaded LV (61). In addition, the RV has higher collagen content than the LV at baseline (75), and there are different patterns of matrix metalloproteinases and extracellular matrix protein-expression patterns between the RV and LV (76).

In summary, there are data that suggest excess RV fibrosis is pathological, but currently available therapies do not effectively reverse RV fibrosis. Of note, these results suggest the RV and LV have distinct fibrotic responses that will require further evaluation to determine how to target RV fibrosis effectively.

### Disrupted transverse tubule architecture

Transverse (t)-tubules are cell membrane invaginations that penetrate into cardiac cells and enhance excitation-contraction coupling (77). In monocrotaline PAH, RV t-tubule structure is deranged, and junctophilin-2 (an essential t-tubule structural protein) (78) expression is reduced (79). Sildenafil increases junctophilin-2 expression, improves t-tubule architecture, and augments RV function however in the setting of reduced RV afterload in monocrotaline rats (79). We also showed t-tubule disarray and downregulation of junctophilin-2 in the monocrotaline RV (80). Colchicine treatment to combat pathological microtubule remodeling increases junctophilin-2, partially corrects t-tubule architecture, and improves RV function in monocrotaline rats, albeit in the setting of less severe pulmonary vascular remodeling (80). These data suggest that t-tubule remodeling promotes RV dysfunction, but both studies have the caveat of reduced PAH severity (79,80). Nonetheless, strategies to normalize junctophilin-2 levels and restore t-tubule architecture may enhance RV function.

### Sarcomeric abnormalities

There is emerging evidence that altered sarcomere function promotes RV dysfunction in PAH. In pulmonary artery-banded rats, RV myocardial stiffness is observed in both mild and severe dysfunction (62). In human studies, PAH patients have increased RV cardiomyocyte sarcomeric stiffness and decreased titin phosphorylation, although there is no difference between the relative expression of the stiff titin isoform N2B compared with the compliant isoform N2BA (63). There is also altered sarcomeric force generation in PAH, as skinned RV cardiomyocytes from patients with SSc-PAH have decreased maximal calcium-activated force and increased calcium sensitivity compared with control (81). Conversely, IPAH sarcomeres exhibit a hypercontractile response and similar calcium sensitivity when compared with controls (81). Importantly, the differences in sarcomeric function can be normalized with protein kinase A (PKA) (81), which suggests PKA activators may combat sarcomeric dysfunction in SSc-PAH. Collectively, these data show that improvement in sarcomere function may be beneficial for patients with PAH and RV dysfunction by enhancing both systolic and diastolic function.

### Inflammation

The role of inflammation in the pathogenesis of pulmonary vascular remodeling is well defined (82,83), but its role in RV dysfunction is not. However, evidence from LV dysfunction preclinical models suggests that anti-inflammatory therapies may augment cardiac function. First, genetic deletion of interleukin (IL)-6 attenuates pressure overload-induced LV hypertrophy and dysfunction in mice (84). Furthermore, cardiac-specific ablation of Ca^2+^/calmodulin-dependent protein kinase II δ decreases activation of the nucleotide-binding oligomerization domain-like receptor pyrin domain-containing protein 3 (NLRP3) inflammasome, decreases macrophage accumulation, and improves LV function in mice (85). Moreover, cardiomyocyte-specific knockout of regnase-1, an RNase involved in degrading proinflammatory cytokine mRNAs, increases IL-6 expression, leading to more severe heart failure following pressure overload in mice (86). Importantly, this phenotype is reversed by either upregulation of regnase-1 expression or administration of an anti-IL-6 receptor antibody (86). Clearly, there is evidence that inflammation negatively affects cardiac function.

Inflammatory mediators are thought to promote adverse RV remodeling and dysfunction (87), which is supported by observations demonstrating an inverse correlation between inflammatory cytokine levels and RV function in PAH. IL-6 levels are independently associated with RV dysfunction in patients with PAH (88), and increased plasma levels of plasma CXC-chemokine ligand (CXCL) 10, CXCL12, and CXCL16 are associated with RV dysfunction in patients with IPAH (89). Macrophages (90), mast cells (91), leukocytes (92), neutrophils (90), and inflammatory cytokines including chemokines (C-C motif chemokine [CCL] 2, CCL5, CXCL6, CXCL9, CXCL10, CXCL12, CXCL13, CXCL16, and C-X3-C motif chemokine ligand 1) (93), tumor necrosis factor-α (90,93), IL-1 (93), IL-6 (and downregulation of IL-10) (93), and nuclear factor kappa B (94) are upregulated in RV pressure overload animal models.

There are inherent differences in the inflammatory cell populations in the LV and RV, suggesting inflammation may have different effects on each ventricle. Mouse studies show the RV has a 4-fold increase in macrophages and dendritic cells at baseline compared with the LV (95). Perhaps pathological inflammation may disproportionately affect the RV, and therefore anti-inflammatory therapies may preferentially augment RV function.

Clinical trials investigating the effects of antagonizing IL-1 (anakinra), IL-6 (tocilizumab), and CD20 on B cells (rituximab) in PAH are completed (Table 3), but not all of the results are available. In an open-label study of 10 patients with PAH, anakinra significantly reduced heart failure symptom burden 2 weeks after treatment, suggesting that it may be beneficial to the RV (96). Certainly, anti-inflammatory therapies are not RV specific, but analysis of hemodynamic changes in these trials may allow us to understand if the advantageous effects are mediated by alterations in the pulmonary vasculature or directly targeting the RV.

### Sex hormones

Numerous preclinical studies show biological sex and sex hormones are major determinants of RV function in RV pressure overload (97). Male Sugen-hypoxia rats have worse RV function than female rats, but oophorectomy worsens RV function in female rats (98), suggesting that estrogen may enhance RV function. Mechanistic studies show exogenous estrogen replacement in ovariectomized female and male rats improves RV function, attenuates RV hypertrophy, and reduces expression of proinflammatory, proapoptotic, angiogenic, and oxidative stress pathways (98). In addition, estrogen preserves RV function by augmenting mitochondrial function by increasing expression of peroxisome proliferator-activated receptor-γ coactivator (PGC)-1α levels in rats (99). Conversely, the male sex hormone, testosterone, is associated with worse RV function in RV pressure overload, as castration attenuates RV hypertrophy and fibrosis and improves survival in pulmonary artery-banded mice (100). Another sex hormone, dehydroepiandrosterone (DHEA), an androgen hormone precursor, may also play a role in RV remodeling, as DHEA restores RV structure and function in Sugen-hypoxia rats, although in the setting of decreased severity of PAH (101). However, in human studies, higher DHEA levels in women are associated with lower RV ejection fraction, and a similar but nonsignificant association is also observed in men (102). The effect of DHEA supplementation on RV function is being investigated in an ongoing clinical trial (Table 3), and this trial will help clarify the direct effects of DHEA on the RV.

In patients with PAH, there is a sex paradox, as PAH is more prevalent in female patients (103), but male sex is associated with higher mortality (104). Importantly, the survival differences can be partially explained by RV function, as male patients have lower RV ejection fraction independent of pulmonary vascular resistance index and LV ejection fraction (105). Thus, clinical studies further support the notion that sex hormones may modulate RV function in PAH.

Preclinical studies imply there are ventricle-specific differences in estrogen signaling. In the dysfunctional rodent RV, the beneficial effects of estrogen are predominately mediated by estrogen receptor (ER)-α (98). Conversely, in LV pressure-overloaded mice, stimulation of ER-β, but not ER-α, normalizes LV ejection fraction (106). Continued studies of the mechanisms by which sex hormones alter RV function are needed to identify pharmaceutical targets in the future.

Of note, there are several ongoing clinical trials evaluating the effects of antagonizing estrogen (Table 3), as higher estradiol levels are associated with the development of pulmonary vascular disease (107). It will be important to determine how these antiestrogen therapies affect RV function.

### Metabolic remodeling and mitochondrial dysfunction

Alterations in metabolism during maladaptive RV remodeling have been extensively reviewed (108,109). In brief, during RV hypertrophy, there is a switch from aerobic to anaerobic metabolism with reduced glucose oxidation; increased uncoupled glycolysis; and enhanced glucose uptake, known as the Warburg effect (108). Several preclinical studies demonstrate that targeting altered metabolism and mitochondrial dysfunction is beneficial to the pressure overloaded RV. The glutamine antagonist, 6-diazo-5-oxo-L-norleucine (DON), decreases glutaminolysis and RV hypertrophy, increases glucose oxidation, and improves cardiac output and exercise capacity in the monocrotaline rat model of PAH (110). Empagliflozin, a sodium-glucose cotransporter 2 (SGLT2) inhibitor, negates pulmonary vascular remodeling, reduces RV hypertrophy and fibrosis, and improves survival in the monocrotaline rat model (111). Interestingly, SGLT2 inhibitors switch fuel use from glucose to free fatty acids, ketone bodies, and branched-chain amino acids in LV failure, which enhances LV function in pigs (112). Perhaps SGLT2 inhibitors have similar beneficial metabolic effects in the RV and can help explain the survival differences in monocrotaline rats.

There are multiple preclinical studies showing alterations in RV metabolism in PAH. Inhibition of fatty acid oxidation (FAO) with ranolazone and trimetazidine increases glucose oxidation, which is advantageous because glucose oxidation is more efficient than fatty acid metabolism (113), and results in improved RV function in pulmonary artery-banded rats (113). However, there are also data showing enhancing FAO can augment RV function. For instance, the peroxisome proliferator-activated receptor γ agonist, pioglitazone normalizes RV FAO gene expression, enhances FAO in isolated RV cardiomyocytes, and prevents RV failure in Sugen-hypoxia rats (114), but this is in the setting of near normalization of PA pressures. Moreover, abnormalities in FAO gene regulation in human RV PAH samples are observed (114), which are likely due to dysregulation of miR-197 and miR-146b. Furthermore, fatty acid metabolites in human PAH RV samples are significantly altered (115). Clearly, FAO is dysregulated in RV failure, but more studies are needed to clearly define how alterations in FAO modulate RV function in PAH. Moreover, bone morphogenetic protein receptor type 2 (BMPR2) mutations alter insulin signaling and glucose metabolism in H9c2 cardiomyocytes (116), which links heritable PAH to metabolic RV derangements. Finally, RV ischemia-reperfusion injury in monocrotaline rats promotes diastolic dysfunction through mitochondrial dysfunction via excess mitochondrial fission (117). Excess mitochondrial fission is mediated by altered regulation of dynamin-related protein 1 and fission protein 1, and inhibition of mitochondrial fission normalizes RV diastolic function (117).

More recent publications highlight additional metabolic deficiencies in the RV in PAH. There is alteration of RV FAO as patients with PAH have significant increases in RV free fatty acids but lower levels of acylcarnitines, which suggests the conversion of fatty acid to acylcarnitines is disrupted in the PAH RV (115). Of note, alterations in fatty acids are not as predominant in dilated cardiomyopathy samples (115), showing a RV-enhanced effect.

Several recent clinical trials studying the FAO inhibitors, ranolazine (118) and trimetazidine, show trends in improvement of RV ejection fraction, but the sample sizes are small (Table 3). Larger trials may be needed to determine the utility of FAO inhibitors in RV dysfunction in PAH.

### RV ischemia

During maladaptive RV remodeling in PAH, RV ischemia results from decreased right coronary artery perfusion pressure or capillary rarefaction. Although the development of RV ischemia from reduced coronary perfusion pressure is well established (119), the role and molecular mechanisms of microvascular ischemia caused by capillary rarefaction and decreased angiogenesis in PAH are less defined and controversial.

A detailed review of the role of angiogenesis during RV failure was previously published (120). In summary, multiple preclinical PAH models have shown decreased RV vascular density during RV failure (110,121, 122, 123). However, a study in Sugen-hypoxia rats challenged those findings, as the authors show that there is actually increased vascular length and volume in the RV, closely proportional to the degree of RV hypertrophy (124). Moreover, in human PAH, there is also increased total vascular length in the RV compared with controls (125). These discrepant findings may be due to differences in the method of histological assessment of capillary density, with possible underestimation of capillary length and surface area with analysis of 2-dimensional sections (126). The molecular mediators of angiogenesis in the RV are incompletely understood, and many of the angiogenic regulators identified in the LV have not yet been studied in the RV. For instance, hypoxia-inducible factors are known to be involved in pulmonary vascular remodeling in PAH (127,128) and in LV remodeling (129,130), but their role in angiogenesis in the RV is uncertain. However, there are multiple angiogenic mediators that are downregulated in maladaptive RV remodeling, including vascular endothelial growth factor (VEGF)-A (121, 122, 123,131); apelin (131,132); and an angiogenic microRNA (miRNA), miR-126 (133). Further studies are required to understand RV angiogenesis.

Several therapies that inhibit capillary rarefaction and restore RV perfusion in preclinical models improve RV function in PAH. The antioxidant Protandim (LifeVantage Corporation, Denver, Colorado) prevents capillary loss and fibrosis and augments RV function in Sugen-hypoxia rats (121). In addition, both carvedilol and metoprolol increase VEGF-A, prevent capillary rarefaction, enhance RV function, and increase survival in Sugen-hypoxia and monocrotaline rats (132). Furthermore, a recent publication shows direct augmentation of RV systolic perfusion via supracoronary aortic banding improves RV function in monocrotaline rats (134). In summary, enhanced RV perfusion augments RV function in preclinical models.

The differences in perfusion between the LV and RV may limit expolating LV findings on the mechanisms of ischemia to the RV. In addition to the RV normally being perfused during both systole and diastole (31), it has a reduced microvascular bed with less tissue perfusion at baseline compared with the LV (135). Furthermore, the RV has different mechanisms to augment cardiomyocyte perfusion compared with the LV, such as increased effect of coronary blood flow and pressure on oxygen demand, greater oxygen extraction reserve, and less effective pressure-flow autoregulation (136).

### RV-specific Epigenetic targets and post-translational modifications

Epigenetic processes are defined as molecules and mechanisms that modulate gene expression independent of changes in DNA sequence (137). Recent publications demonstrate epigenetic modulations are emerging as potential therapeutic targets for PAH (138,139). Similarly, investigations into the role of epigenetics in RV dysfunction are gaining traction and highlighting new mechanisms to target to enhance RV function. The currently defined epigenetic mechanisms that affect RV function can be subdivided into noncoding RNA (miRNA and long noncoding RNA [lncRNA]), histone protein modifications, and DNA methylation.

Murine studies have shown baseline differences in the expression of miRNAs between the RV and LV (140), and multiple candidate miRNAs have been implicated in RV failure, with some evidence they can be manipulated to improve RV function. Although most of the dysregulated miRNAs in RV failure are similar to those found in LV failure, there are miRNAs that are specifically disrupted in RV dysfunction (39,133,140, 141, 142, 143, 144, 145) (Table 1). Of note, miR-197 and miR-146b are upregulated in Sugen-hypoxia and human PAH failing RVs (114). At present, studies on the role of lncRNA in RV failure are only in its infancy. However, a recent manuscript showed the lncRNA H19 is upregulated in the RV of monocrotaline and pulmonary artery-banded rats and human patients with PAH and decompensated RV phenotypes (146). Importantly, silencing H19 expression reduces RVH, fibrosis, and capillary rarefaction and preserves RV function without affecting pulmonary vascular remodeling (146). The therapeutic effects of H19 antagonism are caused by normalization of the histone methyltransferase enhancer of zeste homolog 2 expression (146). Finally, circulating levels of H19 are elevated in patients with IPAH and decompensated RV phenotypes, and H19 levels predict event-free survival rates in a multicenter cohort of patients with PAH (146). Thus, H19 both directly promotes RV dysfunction and is an important biomarker of RV dysfunction that predicts mortality in patients with PAH.

Histones are chromatin proteins that pack DNA (147). Histone acetylation via histone acetyltransferases and deacetylation via histone deacetylases (HDACs) are post-translational modifications that increase and decrease gene transcription, respectively (147). The role of histone modifications on RV function is complex, and currently available data show both beneficial and adverse effects of histone modifications. The pan-HDAC inhibitor, trichostatin A, exacerbates RV dysfunction and remodeling in pulmonary artery-banded rats (148). Alternatively, the Class I HDAC inhibitor, valproic acid impedes the development of RVH in pulmonary artery-banded and monocrotaline rats (149), and the use of another selective Class I HDAC inhibitor, MGCD0103 moderately reduces RVH and inhibits proapoptotic and proinflammatory gene expression in hypoxic rats (150). These data suggest that targeted suppression of Class I HDACs may be beneficial for reversing RV failure, whereas pan-HDAC inhibition may have deleterious effects on the RV.

Although largely unexplored in the RV, DNA methylation differences are observed in the diseased human LV (151,152). However, a recent study links DNA methylation to RV dysfunction via metabolic remodeling. In this study, increased DNA methylation mediated by DNA methyltransferase 1 promotes a profibrotic phenotype in RV fibroblasts, which is associated with more severe RV dysfunction in monocrotaline rats (153). Further studies are needed to understand DNA methylation in RV failure and whether it may be a therapeutic target.

The proteasome is a multicatalytic complex that regulates protein homeostasis via protein degradation (154). The protein post-translational modification, ubiquitination, signals the proteasome to degrade ubiquitinated proteins (155,156). Although not explored in detail, there is evidence of altered proteasome regulation in the RV in PAH. In pulmonary artery-banded mice, there is increased assembly of the 26S proteasome and elevated expression of Rpn6, a subunit involved in 26S proteasome assembly, in the RV. Proteasome inhibition with bortezomib and ONX-0912 mitigates RVH and enhances RV function (157). In contrast, other studies show decreased proteasome activity and increased abundance of ubiquitinated proteins in pulmonary artery-banded (158), monocrotaline (159), and hypoxic RV (159). Furthermore, there is slight improvement of RV function and survival when proteasome function is enhanced via overexpression of the 11S PA28α subunit of the proteasome in mice (158). Certainly, the divergence in proteasome activity could be due to differences in severity of RV dysfunction. Further studies are needed to clarify the intricacies of the proteasome to determine if proteasome modulation may be a therapeutic target for RV failure.

### Exercise and the RV

The causes of impaired exercise capacity in PAH are multifactorial, likely caused by a combination of cardiac (reduced RV function, impaired RV-pulmonary artery coupling, RV ischemia, etc.), pulmonary (increased dead space, increased arterial stiffness and reduced distensibility, respiratory muscle dysfunction, etc.), skeletal muscle, and other systemic limitations (iron deficiency, increased oxidative stress, increased sympathetic activity, etc.) (160). However, supervised exercise training increases exercise capacity in patients with PAH (161), and some of the beneficial effects may be mediated via enhanced RV performance. There are several preclinical and clinical studies suggesting that exercise can improve RV function in PAH. In monocrotaline rats, treadmill training elevated RV capillary density but did not change the amount of RV hypertrophy or fibrosis (92). However, high-intensity interval training specifically increases RV apelin expression while reducing RV fibrosis and RVH in monocrotaline rats (162). Finally, in patients with PAH caused by congenital heart disease, N-terminal-pro-brain natriuretic peptide (NT-proBNP) levels are reduced after 15 weeks of supervised exercise training (163). Collectively, these data suggest that exercise may modulate RV function directly in PAH.

Phosphodiesterase-5 (PDE5) inhibitors increase exercise capacity in patients with PAH (164), and some of the effects are likely mediated via augmented RV function. Although enhancement of RV function by PDE5 inhibitors is largely caused by reduced RV afterload, preclinical studies suggest that PDE5 inhibitors have direct inotropic effects on the RV, as increased cyclic guanosine monophosphate (cGMP) heightens RV contractility in isolated rodent hearts (165). The beneficial effects of PDE5 inhibitors on the RV and exercise capacity are also supported by a randomized controlled trial that investigated the effects of sildenafil (a PDE5 inhibitor) therapy on exercise performance in patients with PH due to LV systolic function (166). In this trial, sildenafil treatment heightened maximal volume of oxygen consumption, exercise cardiac output, and RV ejection fraction at rest and at peak exercise (166). However, the significant reduction in pulmonary vascular resistance needs to be considered when interpreting the change in RV function with sildenafil therapy in these patients.

Certainly, further research is needed to understand the mechanisms by which exercise improves RV function in PAH and other PH subgroups. Several ongoing clinical trials are investigating the role of exercise on RV function (Table 3); it is hoped that they will provide more insight moving forward.

## Targeting the RV in Clinical Trials

Multiple clinical trials have investigated or are currently investigating the targets of RV dysfunction discussed in this review (Table 3). However, majority of these clinical trials are not specifically investigating RV function as a primary outcome. There are some encouraging preliminary findings, as carvedilol enhances RV function (167) and decreases RV glucose uptake (168). In addition, ranolazine (118) and trimetazidine show trends for improvement in RV function. It is hoped that 1 or more of these trials will identify targets for RV failure to improve outcomes in patients with PAH and, eventually, patients with PH due to other causes.

## Conclusions

RV dysfunction is the strongest predictor of mortality in PAH (1, 2, 3), but none of the currently available PAH therapies directly target the failing RV. Here, we discussed the known molecular mechanisms involved in RV dysfunction in PAH to delineate potential targets for RV-enhancing or RV-directed therapies (Central Illustration). Furthermore, we highlighted the important differences between the RV and LV, which may be particularly relevant for developing RV-directed therapies. Finally, we summarized recent and ongoing clinical trials targeting the molecular mechanisms of RV dysfunction in PAH. We hope that ongoing and future studies will identify RV-targeted therapies that will enhance quality of life and improve survival in PAH.

**Central Illustration:**
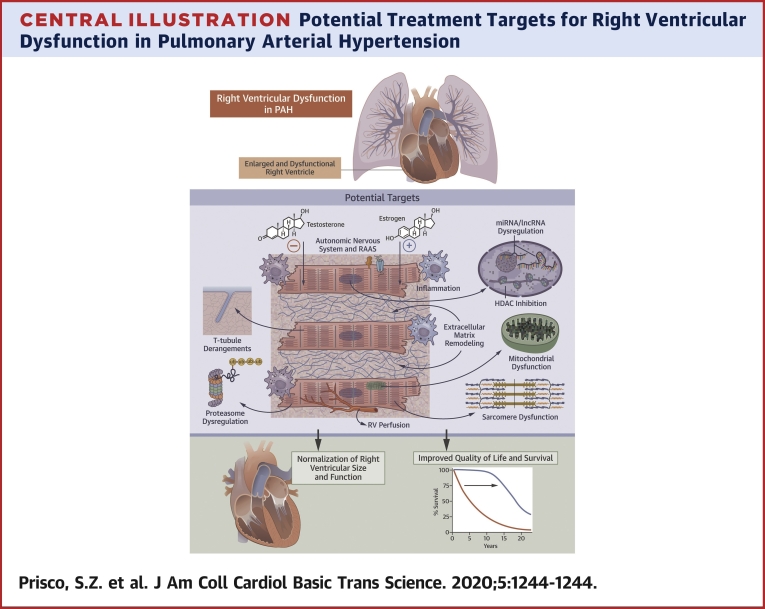
Potential Treatment Targets for Right Ventricular Dysfunction in Pulmonary Arterial Hypertension HDAC = histone deacetylase; lncRNA = long noncoding RNA; miRNA = microRNA; PAH = pulmonary arterial hypertension; RAAS = renin-angiotensin-aldosterone system; RV = right ventricle; t-tubule = transverse tubule; Ub = ubiquitin.

## Author Disclosures

Dr. Prisco is funded by National Institutes of Health (NIH) grant T32 HL144472, a University of Minnesota Clinical and Translational Science award (NIH UL1 TR0029494), and a University of Minnesota Medical School Academic Investment Educational Program grant. Dr. Prins is funded by NIH K08 HL140100, the Jenesis Award from United Therapeutics, a Lillehei Heart Institute Cardiovascular Seed Grant, and the Cardiovascular Medical Research and Education Fund; and has served as a consultant for Actelion and receives grant funding from United Therapeutics. Dr. Thenappan has served as a consultant for Actelion and Gilead. The content of this article is solely the responsibility of the authors and does not represent the official views of the NIH or any other funding sources. All other authors have reported that they have no relationships relevant to the contents of this paper to disclose.

## References

[1] Thenappan T., Shah S.J., Rich S., Tian L., Archer S.L., Gomberg-Maitland M. (2010). Survival in pulmonary arterial hypertension: a reappraisal of the NIH risk stratification equation. Eur Respir J.

[2] Humbert M., Sitbon O., Yaïci A. (2010). Survival in incident and prevalent cohorts of patients with pulmonary arterial hypertension. Eur Respir J.

[3] Benza R.L., Miller D.P., Gomberg-Maitland M. (2010). Predicting survival in pulmonary arterial hypertension: insights from the Registry to Evaluate Early and Long-Term Pulmonary Arterial Hypertension Disease Management (REVEAL). Circulation.

[4] van de Veerdonk M.C., Kind T., Marcus J.T. (2011). Progressive right ventricular dysfunction in patients with pulmonary arterial hypertension responding to therapy. J Am Coll Cardiol.

[5] Mazurek J.A., Vaidya A., Mathai S.C., Roberts J.D., Forfia P.R. (2017). Follow-up tricuspid annular plane systolic excursion predicts survival in pulmonary arterial hypertension. Pulm Circ.

[6] Tedford R.J., Mudd J.O., Girgis R.E. (2013). Right ventricular dysfunction in systemic sclerosis-associated pulmonary arterial hypertension. Circ Heart Fail.

[7] Argula R.G., Karwa A., Lauer A. (2017). Differences in right ventricular functional changes during treatment between systemic sclerosis-associated pulmonary arterial hypertension and idiopathic pulmonary arterial hypertension. Ann Am Thorac Soc.

[8] Hsu S., Houston B.A., Tampakakis E. (2016). Right ventricular functional reserve in pulmonary arterial hypertension. Circulation.

[9] Wijeratne D.T., Lajkosz K., Brogly S.B. (2018). Increasing incidence and prevalence of World Health Organization groups 1 to 4 pulmonary hypertension: a population-based cohort study in Ontario, Canada. Circ Cardiovasc Qual Outcomes.

[10] Strange G., Playford D., Stewart S. (2012). Pulmonary hypertension: prevalence and mortality in the Armadale echocardiography cohort. Heart.

[11] Padang R., Chandrashekar N., Indrabhinduwat M. (2020). Aetiology and outcomes of severe right ventricular dysfunction. Eur Heart J.

[12] Mohammed S.F., Hussain I., AbouEzzeddine O.F. (2014). Right ventricular function in heart failure with preserved ejection fraction: a community-based study. Circulation.

[13] Melenovsky V., Hwang S.J., Lin G., Redfield M.M., Borlaug B.A. (2014). Right heart dysfunction in heart failure with preserved ejection fraction. Eur Heart J.

[14] Bosch L., Lam C.S.P., Gong L. (2017). Right ventricular dysfunction in left-sided heart failure with preserved versus reduced ejection fraction. Eur J Heart Fail.

[15] Prins K.W., Rose L., Archer S.L. (2018). Disproportionate right ventricular dysfunction and poor survival in group 3 pulmonary hypertension. Am J Respir Crit Care Med.

[16] Prins K.W., Rose L., Archer S.L. (2019). Clinical determinants and prognostic implications of right ventricular dysfunction in pulmonary hypertension caused by chronic lung disease. J Am Heart Assoc.

[17] Prins K.W., Duval S., Markowitz J., Pritzker M., Thenappan T. (2017). Chronic use of PAH-specific therapy in World Health Organization Group III Pulmonary Hypertension: a systematic review and meta-analysis. Pulm Circ.

[18] Yancy C.W., Jessup M., Bozkurt B. (2013). 2013 ACCF/AHA guideline for the management of heart failure: a report of the American College of Cardiology Foundation/American Heart Association Task Force on Practice Guidelines. J Am Coll Cardiol.

[19] Yancy C.W., Jessup M., Bozkurt B. (2016). 2016 ACC/AHA/HFSA Focused update on new pharmacological therapy for heart failure: an update of the 2013 ACCF/AHA guideline for the Management of Heart Failure: a report of the American College of Cardiology/American Heart Association Task Force on Clinical Practice Guidelines and the Heart Failure Society of America. J Am Coll Cardiol.

[20] van Campen J.S., de Boer K., van de Veerdonk M.C. (2016). Bisoprolol in idiopathic pulmonary arterial hypertension: an explorative study. Eur Respir J.

[21] Leier C.V., Bambach D., Nelson S. (1983). Captopril in primary pulmonary hypertension. Circulation.

[22] Wagner M., Siddiqui M.A. (2007). Signal transduction in early heart development (II): ventricular chamber specification, trabeculation, and heart valve formation. Exp Biol Med (Maywood).

[23] Verzi M.P., McCulley D.J., De Val S., Dodou E., Black B.L. (2005). The right ventricle, outflow tract, and ventricular septum comprise a restricted expression domain within the secondary/anterior heart field. Dev Biol.

[24] St. John Sutton M.G., Raichlen J.S., Reichek N., Huff D.S. (1984). Quantitative assessment of right and left ventricular growth in the human fetal heart: a pathoanatomic study. Circulation.

[25] Rudolph A.M. (1970). The changes in the circulation after birth: their importance in congenital heart disease. Circulation.

[26] Anversa P., Olivetti G., Loud A.V. (1980). Morphometric study of early postnatal development in the left and right ventricular myocardium of the rat. I. Hypertrophy, hyperplasia, and binucleation of myocytes. Circ Res.

[27] Friedberg M.K., Redington A.N. (2014). Right versus left ventricular failure: differences, similarities, and interactions. Circulation.

[28] Brown S.B., Raina A., Katz D., Szerlip M., Wiegers S.E., Forfia P.R. (2011). Longitudinal shortening accounts for the majority of right ventricular contraction and improves after pulmonary vasodilator therapy in normal subjects and patients with pulmonary arterial hypertension. Chest.

[29] Song J.K. (2009). How does the left ventricle work? Ventricular rotation as a new index of cardiac performance. Korean Circ J.

[30] Maciver D.H. (2012). The relative impact of circumferential and longitudinal shortening on left ventricular ejection fraction and stroke volume. Exp Clin Cardiol.

[31] Crystal G.J., Pagel P.S. (2018). Right Ventricular perfusion: physiology and clinical implications. Anesthesiology.

[32] Marcus M., Wright C., Doty D. (1981). Measurements of coronary velocity and reactive hyperemia in the coronary circulation of humans. Circ Res.

[33] Kusachi S., Nishiyama O., Yasuhara K., Saito D., Haraoka S., Nagashima H. (1982). Right and left ventricular oxygen metabolism in open-chest dogs. Am J Physiol.

[34] Crystal G.J., Kim S.J., Salem M.R. (1993). Right and left ventricular O2 uptake during hemodilution and beta-adrenergic stimulation. Am J Physiol.

[35] Zong P., Sun W., Setty S., Tune J.D., Downey H.F. (2004). Alpha-adrenergic vasoconstrictor tone limits right coronary blood flow in exercising dogs. Exp Biol Med (Maywood).

[36] Naeije R., Brimioulle S., Dewachter L. (2014). Biomechanics of the right ventricle in health and disease (2013 Grover Conference series). Pulm Circ.

[37] Müller-Strahl G., Hemker J., Zimmer H.G. (2002). Comparison between left and right heart function in the isolated biventricular working rat heart. Exp Clin Cardiol.

[38] Taverne Y.J.H.J., Sadeghi A., Bartelds B., Bogers A.J.J.C., Merkus D. (2020). Right ventricular phenotype, function, and failure: a journey from evolution to clinics. Heart Fail Rev.

[39] Reddy S., Bernstein D. (2015). Molecular mechanisms of right ventricular failure. Circulation.

[40] de Lucia C., Eguchi A., Koch W.J. (2018). New insights in cardiac β-adrenergic signaling during heart failure and aging. Front Pharmacol.

[41] Woodcock E.A., Du X.J., Reichelt M.E., Graham R.M. (2008). Cardiac alpha 1-adrenergic drive in pathological remodelling. Cardiovasc Res.

[42] Lowes B.D., Minobe W., Abraham W.T. (1997). Changes in gene expression in the intact human heart. Downregulation of alpha-myosin heavy chain in hypertrophied, failing ventricular myocardium. J Clin Invest.

[43] Dechesne C.A., Leger J.O., Leger J.J. (1987). Distribution of alpha- and beta-myosin heavy chains in the ventricular fibers of the postnatal developing rat. Dev Biol.

[44] Rich S., Seidlitz M., Dodin E. (1998). The short-term effects of digoxin in patients with right ventricular dysfunction from pulmonary hypertension. Chest.

[45] Gheorghiade M., Adams K.F., Colucci W.S. (2004). Digoxin in the management of cardiovascular disorders. Circulation.

[46] Lahm T., Douglas I.S., Archer S.L. (2018). Assessment of right ventricular function in the research setting: knowledge gaps and pathways forward. an official American Thoracic Society Research Statement. Am J Respir Crit Care Med.

[47] Perros F., de Man F.S., Bogaard H.J. (2017). Use of β-blockers in pulmonary hypertension. Circ Heart Fail.

[48] Yoshida K., Saku K., Kamada K. (2018). Electrical vagal nerve stimulation ameliorates pulmonary vascular remodeling and improves survival in rats with severe pulmonary arterial hypertension. J Am Coll Cardiol Basic Trans Science.

[49] Maron B.A., Leopold J.A. (2015). Emerging concepts in the molecular basis of pulmonary arterial hypertension: part ii: neurohormonal signaling contributes to the pulmonary vascular and right ventricular pathophenotype of pulmonary arterial hypertension. Circulation.

[50] Iusuf D., Henning R.H., van Gilst W.H., Roks A.J. (2008). Angiotensin-(1-7): pharmacological properties and pharmacotherapeutic perspectives. Eur J Pharmacol.

[51] Shenoy V., Ferreira A.J., Qi Y. (2010). The angiotensin-converting enzyme 2/angiogenesis-(1-7)/Mas axis confers cardiopulmonary protection against lung fibrosis and pulmonary hypertension. Am J Respir Crit Care Med.

[52] Okada M., Kikuzuki R., Harada T., Hori Y., Yamawaki H., Hara Y. (2008). Captopril attenuates matrix metalloproteinase-2 and -9 in monocrotaline-induced right ventricular hypertrophy in rats. J Pharmacol Sci.

[53] Rouleau J.L., Kapuku G., Pelletier S. (2001). Cardioprotective effects of ramipril and losartan in right ventricular pressure overload in the rabbit: importance of kinins and influence on angiotensin II type 1 receptor signaling pathway. Circulation.

[54] Okada M., Harada T., Kikuzuki R., Yamawaki H., Hara Y. (2009). Effects of telmisartan on right ventricular remodeling induced by monocrotaline in rats. J Pharmacol Sci.

[55] Rondelet B., Kerbaul F., Van Beneden R. (2005). Prevention of pulmonary vascular remodeling and of decreased BMPR-2 expression by losartan therapy in shunt-induced pulmonary hypertension. Am J Physiol Heart Circ Physiol.

[56] Maron B.A., Zhang Y.Y., White K. (2012). Aldosterone inactivates the endothelin-B receptor via a cysteinyl thiol redox switch to decrease pulmonary endothelial nitric oxide levels and modulate pulmonary arterial hypertension. Circulation.

[57] Boehm M., Arnold N., Braithwaite A. (2018). Eplerenone attenuates pathological pulmonary vascular rather than right ventricular remodeling in pulmonary arterial hypertension. BMC Pulm Med.

[58] Zisman L.S., Asano K., Dutcher D.L. (1998). Differential regulation of cardiac angiotensin converting enzyme binding sites and AT1 receptor density in the failing human heart. Circulation.

[59] Abraham W.T., Raynolds M.V., Badesch D.B. (2003). Angiotensin-converting enzyme DD genotype in patients with primary pulmonary hypertension: increased frequency and association with preserved haemodynamics. J Renin Angiotensin Aldosterone Syst.

[60] Hemnes A.R., Rathinasabapathy A., Austin E.A. (2018). A potential therapeutic role for angiotensin-converting enzyme 2 in human pulmonary arterial hypertension. Eur Respir J.

[61] Andersen S., Nielsen-Kudsk J.E., Vonk Noordegraaf A., de Man F.S. (2019). Right ventricular fibrosis. Circulation.

[62] Rain S., Andersen S., Najafi A. (2016). Right ventricular myocardial stiffness in experimental pulmonary arterial hypertension: relative contribution of fibrosis and myofibril stiffness. Circ Heart Fail.

[63] Rain S., Handoko M.L., Trip P. (2013). Right ventricular diastolic impairment in patients with pulmonary arterial hypertension. Circulation.

[64] Kusakari Y., Urashima T., Shimura D. (2017). Impairment of Excitation-contraction coupling in right ventricular hypertrophied muscle with fibrosis induced by pulmonary artery banding. PLoS One.

[65] Gomez-Arroyo J., Sakagami M., Syed A.A. (2015). Iloprost reverses established fibrosis in experimental right ventricular failure. Eur Respir J.

[66] Choudhary G., Troncales F., Martin D., Harrington E.O., Klinger J.R. (2011). Bosentan attenuates right ventricular hypertrophy and fibrosis in normobaric hypoxia model of pulmonary hypertension. J Heart Lung Transplant.

[67] Friedberg M.K., Cho M.Y., Li J. (2013). Adverse biventricular remodeling in isolated right ventricular hypertension is mediated by increased transforming growth factor-β1 signaling and is abrogated by angiotensin receptor blockade. Am J Respir Cell Mol Biol.

[68] Yung L.M., Nikolic I., Paskin-Flerlage S.D., Pearsall R.S., Kumar R., Yu P.B. (2016). A selective transforming growth factor-β ligand trap attenuates pulmonary hypertension. Am J Respir Crit Care Med.

[69] Janssen W., Schymura Y., Novoyatleva T. (2015). 5-HT2B receptor antagonists inhibit fibrosis and protect from RV heart failure. Biomed Res Int.

[70] Boehm M., Tian X., Mao Y. (2020). Delineating the molecular and histological events that govern right ventricular recovery using a novel mouse model of PA de-banding. Cardiovasc Res.

[71] Yamagami K., Oka T., Wang Q. (2015). Pirfenidone exhibits cardioprotective effects by regulating myocardial fibrosis and vascular permeability in pressure-overloaded hearts. Am J Physiol Heart Circ Physiol.

[72] Andersen S., Birkmose Axelsen J., Ringgaard S. (2019). Pressure overload induced right ventricular remodeling is not attenuated by the anti-fibrotic agent pirfenidone. Pulm Circ.

[73] Poble P.B., Phan C., Quatremare T. (2019). Therapeutic effect of pirfenidone in the sugen/hypoxia rat model of severe pulmonary hypertension. FASEB J.

[74] Zhang Q., Saito Y., Naya N. (2008). The specific mineralocorticoid receptor blocker eplerenone attenuates left ventricular remodeling in mice lacking the gene encoding guanylyl cyclase-A. Hypertens Res.

[75] Oken D.E., Boucek R.J. (1957). Quantitation of collagen in human myocardium. Circ Res.

[76] Herpel E., Singer S., Flechtenmacher C. (2005). Extracellular matrix proteins and matrix metalloproteinases differ between various right and left ventricular sites in end-stage cardiomyopathies. Virchows Arch.

[77] Ibrahim M., Gorelik J., Yacoub M.H., Terracciano C.M. (2011). The structure and function of cardiac t-tubules in health and disease. Proc Biol Sci.

[78] Takeshima H., Komazaki S., Nishi M., Iino M., Kangawa K. (2000). Junctophilins: a novel family of junctional membrane complex proteins. Mol Cell.

[79] Xie Y.P., Chen B., Sanders P. (2012). Sildenafil prevents and reverses transverse-tubule remodeling and Ca(2+) handling dysfunction in right ventricle failure induced by pulmonary artery hypertension. Hypertension.

[80] Prins K.W., Tian L., Wu D., Thenappan T., Metzger J.M., Archer S.L. (2017). Colchicine depolymerizes microtubules, increases junctophilin-2, and improves right ventricular function in experimental pulmonary arterial hypertension. J Am Heart Assoc.

[81] Hsu S., Kokkonen-Simon K.M., Kirk J.A. (2018). Right ventricular myofilament functional differences in humans with systemic sclerosis-associated versus idiopathic pulmonary arterial hypertension. Circulation.

[82] Price L.C., Wort S.J., Perros F. (2012). Inflammation in pulmonary arterial hypertension. Chest.

[83] Hassoun P.M., Mouthon L., Barberà J.A. (2009). Inflammation, growth factors, and pulmonary vascular remodeling. J Am Coll Cardiol.

[84] Zhao L., Cheng G., Jin R. (2016). Deletion of interleukin-6 attenuates pressure overload-induced left ventricular hypertrophy and dysfunction. Circ Res.

[85] Suetomi T., Willeford A., Brand C.S. (2018). Inflammation and NLRP3 inflammasome activation initiated in response to pressure overload by Ca. Circulation.

[86] Omiya S., Omori Y., Taneike M. (2020). Cytokine mRNA degradation in cardiomyocytes restrains sterile inflammation in pressure-overloaded hearts. Circulation.

[87] Sydykov A., Mamazhakypov A., Petrovic A. (2018). Inflammatory mediators drive adverse right ventricular remodeling and dysfunction and serve as potential biomarkers. Front Physiol.

[88] Prins K.W., Archer S.L., Pritzker M. (2018). Interleukin-6 is independently associated with right ventricular function in pulmonary arterial hypertension. J Heart Lung Transplant.

[89] Yang T., Li Z.N., Chen G. (2014). Increased levels of plasma CXC-chemokine ligand 10, 12 and 16 are associated with right ventricular function in patients with idiopathic pulmonary arterial hypertension. Heart Lung.

[90] Campian M.E., Hardziyenka M., de Bruin K. (2010). Early inflammatory response during the development of right ventricular heart failure in a rat model. Eur J Heart Fail.

[91] Olivetti G., Lagrasta C., Ricci R., Sonnenblick E.H., Capasso J.M., Anversa P. (1989). Long-term pressure-induced cardiac hypertrophy: capillary and mast cell proliferation. Am J Physiol.

[92] Handoko M.L., de Man F.S., Happé C.M. (2009). Opposite effects of training in rats with stable and progressive pulmonary hypertension. Circulation.

[93] Dewachter C., Dewachter L., Rondelet B. (2010). Activation of apoptotic pathways in experimental acute afterload-induced right ventricular failure. Crit Care Med.

[94] Nogueira-Ferreira R., Moreira-Gonçalves D., Silva A.F. (2016). Exercise preconditioning prevents MCT-induced right ventricle remodeling through the regulation of TNF superfamily cytokines. Int J Cardiol.

[95] Gorr M.W., Sriram K., Chinn A.M., Muthusamy A., Insel P.A. (2020). Transcriptomic profiles reveal differences between the right and left ventricle in normoxia and hypoxia. Physiol Rep.

[96] Trankle C.R., Canada J.M., Kadariya D. (2019). IL-1 Blockade reduces inflammation in pulmonary arterial hypertension and right ventricular failure: a single-arm, open-label, phase IB/II pilot study. Am J Respir Crit Care Med.

[97] Hester J., Ventetuolo C., Lahm T. (2019). Sex, gender, and sex hormones in pulmonary hypertension and right ventricular failure. Compr Physiol.

[98] Frump A.L., Goss K.N., Vayl A. (2015). Estradiol improves right ventricular function in rats with severe angioproliferative pulmonary hypertension: effects of endogenous and exogenous sex hormones. Am J Physiol Lung Cell Mol Physiol.

[99] Liu A., Philip J., Vinnakota K.C. (2017). Estrogen maintains mitochondrial content and function in the right ventricle of rats with pulmonary hypertension. Physiol Rep.

[100] Hemnes A.R., Maynard K.B., Champion H.C. (2012). Testosterone negatively regulates right ventricular load stress responses in mice. Pulm Circ.

[101] Alzoubi A., Toba M., Abe K. (2013). Dehydroepiandrosterone restores right ventricular structure and function in rats with severe pulmonary arterial hypertension. Am J Physiol Heart Circ Physiol.

[102] Ventetuolo C.E., Ouyang P., Bluemke D.A. (2011). Sex hormones are associated with right ventricular structure and function: the MESA-right ventricle study. Am J Respir Crit Care Med.

[103] Badesch D.B., Raskob G.E., Elliott C.G. (2010). Pulmonary arterial hypertension: baseline characteristics from the REVEAL Registry. Chest.

[104] Humbert M., Sitbon O., Chaouat A. (2010). Survival in patients with idiopathic, familial, and anorexigen-associated pulmonary arterial hypertension in the modern management era. Circulation.

[105] Kawut S.M., Al-Naamani N., Agerstrand C. (2009). Determinants of right ventricular ejection fraction in pulmonary arterial hypertension. Chest.

[106] Iorga A., Umar S., Ruffenach G. (2018). Estrogen rescues heart failure through estrogen receptor beta activation. Biol Sex Differ.

[107] Ventetuolo C.E., Baird G.L., Barr R.G. (2016). Higher estradiol and lower dehydroepiandrosterone-sulfate levels are associated with pulmonary arterial hypertension in men. Am J Respir Crit Care Med.

[108] Ryan J.J., Archer S.L. (2014). The right ventricle in pulmonary arterial hypertension: disorders of metabolism, angiogenesis and adrenergic signaling in right ventricular failure. Circ Res.

[109] Ryan J.J., Archer S.L. (2015). Emerging concepts in the molecular basis of pulmonary arterial hypertension: part I: metabolic plasticity and mitochondrial dynamics in the pulmonary circulation and right ventricle in pulmonary arterial hypertension. Circulation.

[110] Piao L., Fang Y.H., Parikh K., Ryan J.J., Toth P.T., Archer S.L. (2013). Cardiac glutaminolysis: a maladaptive cancer metabolism pathway in the right ventricle in pulmonary hypertension. J Mol Med (Berl).

[111] Chowdhury B., Luu A.Z., Luu V.Z. (2020). The SGLT2 inhibitor empagliflozin reduces mortality and prevents progression in experimental pulmonary hypertension. Biochem Biophys Res Commun.

[112] Santos-Gallego C.G., Requena-Ibanez J.A., San Antonio R. (2019). Empagliflozin ameliorates adverse left ventricular remodeling in nondiabetic heart failure by enhancing myocardial energetics. J Am Coll Cardiol.

[113] Fang Y.H., Piao L., Hong Z. (2012). Therapeutic inhibition of fatty acid oxidation in right ventricular hypertrophy: exploiting Randle's cycle. J Mol Med (Berl).

[114] Legchenko E., Chouvarine P., Borchert P. (2018). PPARγ agonist pioglitazone reverses pulmonary hypertension and prevents right heart failure via fatty acid oxidation. Sci Transl Med.

[115] Brittain E.L., Talati M., Fessel J.P. (2016). Fatty acid metabolic defects and right ventricular lipotoxicity in human pulmonary arterial hypertension. Circulation.

[116] Hemnes A.R., Fessel J.P., Chen X. (2020). BMPR2 dysfunction impairs insulin signaling and glucose homeostasis in cardiomyocytes. Am J Physiol Lung Cell Mol Physiol.

[117] Tian L., Neuber-Hess M., Mewburn J. (2017). Ischemia-induced Drp1 and Fis1-mediated mitochondrial fission and right ventricular dysfunction in pulmonary hypertension. J Mol Med (Berl).

[118] Khan S.S., Cuttica M.J., Beussink-Nelson L. (2015). Effects of ranolazine on exercise capacity, right ventricular indices, and hemodynamic characteristics in pulmonary arterial hypertension: a pilot study. Pulm Circ.

[119] van Wolferen S.A., Marcus J.T., Westerhof N. (2008). Right coronary artery flow impairment in patients with pulmonary hypertension. Eur Heart J.

[120] Frump A.L., Bonnet S., de Jesus Perez V.A., Lahm T. (2018). Emerging role of angiogenesis in adaptive and maladaptive right ventricular remodeling in pulmonary hypertension. Am J Physiol Lung Cell Mol Physiol.

[121] Bogaard H.J., Natarajan R., Henderson S.C. (2009). Chronic pulmonary artery pressure elevation is insufficient to explain right heart failure. Circulation.

[122] Partovian C., Adnot S., Eddahibi S. (1998). Heart and lung VEGF mRNA expression in rats with monocrotaline- or hypoxia-induced pulmonary hypertension. Am J Physiol.

[123] Sutendra G., Dromparis P., Paulin R. (2013). A metabolic remodeling in right ventricular hypertrophy is associated with decreased angiogenesis and a transition from a compensated to a decompensated state in pulmonary hypertension. J Mol Med (Berl).

[124] Graham B.B., Kumar R., Mickael C. (2018). Vascular adaptation of the right ventricle in experimental pulmonary hypertension. Am J Respir Cell Mol Biol.

[125] Graham B.B., Koyanagi D., Kandasamy B., Tuder R.M. (2017). Right ventricle vasculature in human pulmonary hypertension assessed by stereology. Am J Respir Crit Care Med.

[126] Hudlicka O., Brown M., Egginton S. (1992). Angiogenesis in skeletal and cardiac muscle. Physiol Rev.

[127] Shimoda L.A., Semenza G.L. (2011). HIF and the lung: role of hypoxia-inducible factors in pulmonary development and disease. Am J Respir Crit Care Med.

[128] Veith C., Schermuly R.T., Brandes R.P., Weissmann N. (2016). Molecular mechanisms of hypoxia-inducible factor-induced pulmonary arterial smooth muscle cell alterations in pulmonary hypertension. J Physiol.

[129] Huang Y., Hickey R.P., Yeh J.L. (2004). Cardiac myocyte-specific HIF-1alpha deletion alters vascularization, energy availability, calcium flux, and contractility in the normoxic heart. FASEB J.

[130] Kido M., Du L., Sullivan C.C. (2005). Hypoxia-inducible factor 1-alpha reduces infarction and attenuates progression of cardiac dysfunction after myocardial infarction in the mouse. J Am Coll Cardiol.

[131] Drake J.I., Bogaard H.J., Mizuno S. (2011). Molecular signature of a right heart failure program in chronic severe pulmonary hypertension. Am J Respir Cell Mol Biol.

[132] Bogaard H.J., Natarajan R., Mizuno S. (2010). Adrenergic receptor blockade reverses right heart remodeling and dysfunction in pulmonary hypertensive rats. Am J Respir Crit Care Med.

[133] Potus F., Ruffenach G., Dahou A. (2015). Downregulation of microRNA-126 contributes to the failing right ventricle in pulmonary arterial hypertension. Circulation.

[134] Tian L., Xiong P.Y., Alizadeh E. (2020). Supra-coronary aortic banding improves right ventricular function in experimental pulmonary arterial hypertension in rats by increasing systolic right coronary artery perfusion. Acta Physiol (Oxf).

[135] Ohuchi H., Beighley P.E., Dong Y., Zamir M., Ritman E.L. (2007). Microvascular development in porcine right and left ventricular walls. Pediatr Res.

[136] Zong P., Tune J.D., Downey H.F. (2005). Mechanisms of oxygen demand/supply balance in the right ventricle. Exp Biol Med (Maywood).

[137] Cavalli G., Heard E. (2019). Advances in epigenetics link genetics to the environment and disease. Nature.

[138] Huston J.H., Ryan J.J. (2016). The emerging role of epigenetics in pulmonary arterial hypertension: an important avenue for clinical trials (2015 Grover Conference Series). Pulm Circ.

[139] Gamen E., Seeger W., Pullamsetti S.S. (2016). The emerging role of epigenetics in pulmonary hypertension. Eur Respir J.

[140] Reddy S., Zhao M., Hu D.Q. (2012). Dynamic microRNA expression during the transition from right ventricular hypertrophy to failure. Physiol Genomics.

[141] Powers J.C., Sabri A., Al-Bataineh D. (2020). Differential microRNA-21 and microRNA-221 upregulation in the biventricular failing heart reveals distinct stress responses of right versus left ventricular fibroblasts. Circ Heart Fail.

[142] Potus F., Malenfant S., Graydon C. (2014). Impaired angiogenesis and peripheral muscle microcirculation loss contribute to exercise intolerance in pulmonary arterial hypertension. Am J Respir Crit Care Med.

[143] Paulin R., Sutendra G., Gurtu V. (2015). A miR-208-Mef2 axis drives the decompensation of right ventricular function in pulmonary hypertension. Circ Res.

[144] Joshi S.R., Dhagia V., Gairhe S., Edwards J.G., McMurtry I.F., Gupte S.A. (2016). MicroRNA-140 is elevated and mitofusin-1 is downregulated in the right ventricle of the Sugen5416/hypoxia/normoxia model of pulmonary arterial hypertension. Am J Physiol Heart Circ Physiol.

[145] Chouvarine P., Legchenko E., Geldner J., Riehle C., Hansmann G. (2019). Hypoxia drives cardiac miRNAs and inflammation in the right and left ventricle. J Mol Med (Berl).

[146] Omura J., Habbout K., Shimauchi T. (2020). Identification of the long non-coding RNA H19 as a new biomarker and therapeutic target in right ventricular failure in pulmonary arterial hypertension. Circulation.

[147] Ito K., J Barnes P., M Adcock I. (2000). Histone acetylation and deacetylation. Methods Mol Med.

[148] Bogaard H.J., Mizuno S., Hussaini A.A. (2011). Suppression of histone deacetylases worsens right ventricular dysfunction after pulmonary artery banding in rats. Am J Respir Crit Care Med.

[149] Cho Y.K., Eom G.H., Kee H.J. (2010). Sodium valproate, a histone deacetylase inhibitor, but not captopril, prevents right ventricular hypertrophy in rats. Circ J.

[150] Cavasin M.A., Demos-Davies K., Horn T.R. (2012). Selective class I histone deacetylase inhibition suppresses hypoxia-induced cardiopulmonary remodeling through an antiproliferative mechanism. Circ Res.

[151] Haas J., Frese K.S., Park Y.J. (2013). Alterations in cardiac DNA methylation in human dilated cardiomyopathy. EMBO Mol Med.

[152] Movassagh M., Choy M.K., Knowles D.A. (2011). Distinct epigenomic features in end-stage failing human hearts. Circulation.

[153] Tian L., Wu D., Dasgupta A. (2020). Epigenetic metabolic reprogramming of right ventricular fibroblasts in pulmonary arterial hypertension: a pyruvate dehydrogenase kinase-dependent shift in mitochondrial metabolism promotes right ventricular fibrosis. Circ Res.

[154] Gomes A.V., Young G.W., Wang Y. (2009). Contrasting proteome biology and functional heterogeneity of the 20 S proteasome complexes in mammalian tissues. Mol Cell Proteomics.

[155] Zong C., Gomes A.V., Drews O. (2006). Regulation of murine cardiac 20S proteasomes: role of associating partners. Circ Res.

[156] Zong C., Young G.W., Wang Y. (2008). Two-dimensional electrophoresis-based characterization of post-translational modifications of mammalian 20S proteasome complexes. Proteomics.

[157] Heitmeier T., Sydykov A., Lukas C. (2020). Altered proteasome function in right ventricular hypertrophy. Cardiovasc Res.

[158] Rajagopalan V., Zhao M., Reddy S. (2013). Altered ubiquitin-proteasome signaling in right ventricular hypertrophy and failure. Am J Physiol Heart Circ Physiol.

[159] Fessart D., Martin-Negrier M.L., Claverol S. (2014). Proteomic remodeling of proteasome in right heart failure. J Mol Cell Cardiol.

[160] Tran D.L., Lau E.M.T., Celermajer D.S., Davis G.M., Cordina R. (2018). Pathophysiology of exercise intolerance in pulmonary arterial hypertension. Respirology.

[161] Pandey A., Garg S., Khunger M., Kumbhani D.J., Chin K.M., Berry J.D. (2015). Efficacy and safety of exercise training in chronic pulmonary hypertension: systematic review and meta-analysis. Circ Heart Fail.

[162] Brown M.B., Neves E., Long G. (2017). High-intensity interval training, but not continuous training, reverses right ventricular hypertrophy and dysfunction in a rat model of pulmonary hypertension. Am J Physiol Regul Integr Comp Physiol.

[163] Becker-Grünig T., Klose H., Ehlken N. (2013). Efficacy of exercise training in pulmonary arterial hypertension associated with congenital heart disease. Int J Cardiol.

[164] Wilkins M.R., Wharton J., Grimminger F., Ghofrani H.A. (2008). Phosphodiesterase inhibitors for the treatment of pulmonary hypertension. Eur Respir J.

[165] Nagendran J., Archer S.L., Soliman D. (2007). Phosphodiesterase type 5 is highly expressed in the hypertrophied human right ventricle, and acute inhibition of phosphodiesterase type 5 improves contractility. Circulation.

[166] Lewis G.D., Shah R., Shahzad K. (2007). Sildenafil improves exercise capacity and quality of life in patients with systolic heart failure and secondary pulmonary hypertension. Circulation.

[167] Grinnan D., Bogaard H.J., Grizzard J. (2014). Treatment of group I pulmonary arterial hypertension with carvedilol is safe. Am J Respir Crit Care Med.

[168] Farha S., Saygin D., Park M.M. (2017). Pulmonary arterial hypertension treatment with carvedilol for heart failure: a randomized controlled trial. JCI Insight.

[169] Guihaire J., Bogaard H.J., Flécher E. (2013). Experimental models of right heart failure: a window for translational research in pulmonary hypertension. Semin Respir Crit Care Med.

[170] Stenmark K.R., Meyrick B., Galie N., Mooi W.J., McMurtry I.F. (2009). Animal models of pulmonary arterial hypertension: the hope for etiological discovery and pharmacological cure. Am J Physiol Lung Cell Mol Physiol.

[171] Kawut S.M., Archer-Chicko C.L., DeMichele A. (2017). Anastrozole in pulmonary arterial hypertension: a randomized, double-blind, placebo-controlled trial. Am J Respir Crit Care Med.

[172] Kawut S.M., Pinder D., Al-Naamani N. (2019). Fulvestrant for the Treatment of Pulmonary Arterial Hypertension. Ann Am Thorac Soc.

[173] Gomberg-Maitland M., Schilz R., Mediratta A. (2015). Phase I safety study of ranolazine in pulmonary arterial hypertension. Pulm Circ.

[174] Han Y., Forfia P.R., Vaidya A. (2018). Rationale and design of the ranolazine PH-RV study: a multicentered randomised and placebo-controlled study of ranolazine to improve RV function in patients with non-group 2 pulmonary hypertension. Open Heart.

